# Selective use of postoperative neck radiotherapy in oral cavity and oropharynx cancer: a prospective clinical study

**DOI:** 10.1186/1748-717X-8-103

**Published:** 2013-04-28

**Authors:** Miguel Martínez Carrillo, Isabel Tovar Martín, Ildefonso Martínez Lara, José Mariano Ruiz de Almodóvar Rivera, Rosario Del Moral Ávila

**Affiliations:** 1 Department of Radiation Oncology, Virgen de las Nieves University Hospital, Avda. Fuerzas Armadas 4, Granada 18014, Spain; 2 Department of Maxillo-Facial Surgery, Virgen de las Nieves University Hospital, Granada, Spain; 3 Department of Radiology and Physical Medicine, University of Granada, Granada, Spain

**Keywords:** Oral cavity and oropharynx cancer, Postoperative radiotherapy, Selective neck irradiation, Lymph node

## Abstract

**Background:**

In cervical postoperative radiotherapy, the target volume is usually the same as the extension of the previous dissection. We evaluated a protocol of selective irradiation according to the risk estimated for each dissected lymph node level.

**Methods:**

Eighty patients with oral/oropharyngeal cancer were included in this prospective clinical study between 2005 and 2008. Patients underwent surgery of the primary tumor and cervical dissection, with identification of positive nodal levels, followed by selective postoperative radiotherapy. Three types of selective nodal clinical target volume (CTV) were defined: CTV0, CTV1, and CTV2, with a subclinical disease risk of <10%, 10-25%, and 25% and a prescribed radiation dose of <35 Gy, 50 Gy, and 66–70 Gy, respectively. The localization of node failure was categorized as field, marginal, or outside the irradiated field.

**Results:**

A consistent pattern of cervical infiltration was observed in 97% of positive dissections. Lymph node failure occurred within a high-risk irradiated area (CTV1-CTV2) in 12 patients, marginal area (CTV1/CTVO) in 1 patient, and non-irradiated low-risk area (CTV0) in 2 patients. The volume of selective lymph node irradiation was below the standard radiation volume in 33 patients (mean of 118.6 cc per patient). This decrease in irradiated volume was associated with greater treatment compliance and reduced secondary toxicity. The three-year actuarial nodal control rate was 80%.

**Conclusion:**

This selective postoperative neck irradiation protocol was associated with a similar failure pattern to that observed after standard neck irradiation and achieved a significant reduction in target volume and secondary toxicity.

## Introduction

Squamous cell cancer of the head and neck is diagnosed in 650,000 individuals annually worldwide and accounts for 5-10% of all malignant tumors [1]. The region with the highest incidence is Southern Europe, where there has been a gradual reduction in oral cancer and an increase in oropharyngeal cancer [2].

Squamous cell cancer of the head and neck follows a consistent and well-defined locoregional growth pattern. The therapeutic decision depends on the lymph node metastatic status, because tumor control requires treatment of both the primary tumor and involved regional nodes [3].

Standard guidelines for selecting target volumes are based on knowledge of neck tumor distribution and dissemination patterns. The Memorial Sloan-Kettering Cancer Center described the pattern of cervical lymph node metastasis in 1077 naïve patients with head and neck cancer after performing 1118 neck dissections [4-6]. Neck dissection was performed in 343 of the 341 patients with a clinically N0 neck cancer, i.e., bilateral dissection was only performed in two patients. Bilateral neck dissection was carried out in 39 of the 736 patients with clinically positive nodes. Metastatic disease was confirmed in 33% of elective neck dissections and 82% of therapeutic neck dissections. The distribution of pathologically confirmed metastatic lymph nodes varied according to the primary tumor site. In clinically N0 patients, metastatic lymph nodes were generally observed in levels I-III for oral cavity tumors (20%, 17% and 9% respectively) and in levels II-IV for oropharyngeal tumors (25%, 19% and 8% respectively). A similar pattern was observed in the patients who underwent therapeutic neck dissection except for a higher density of adenopathies and significant pathologic infiltration of an additional level; among tumors of the oral cavity, 44% were in level I, 32% in level II, 16% in level III, and 3% in levels IV; in oropharyngeal tumors, 15% were in level I, 71% in level II, 42% in level III, 27% in level IV, and 9% in level V. Pathological infiltration of level V was low, observing a single infiltration of level V in only one patient; it was below 1% when a single pathologically confirmed positive node was detected in levels I-III but increased to 16% when a single positive node was located in level IV. When more than one level was infiltrated, the likelihood of level V involvement progressively increased, reaching 40% when levels I-IV were all involved. Pathological involvement of level I was observed in only 2% of clinical N0 patients with oropharyngeal tumors, increasing to 15% after therapeutic dissection. These observations illustrate the gradual and orderly infiltration of node levels in the neck.

Surgeons were the first to use the concept of selective treatment, performing selective dissections of the neck to remove only high-risk lymph node levels. This practice led surgeons to divide the neck into different levels according to anatomical references that can be easily identified during neck dissection procedures (i.e. major vessels, muscles, cartilages), known as the Robbins classification [7,8]. Selective neck dissection is currently the procedure of choice for elective neck surgery and for a specific group of neck-positive patients (selected N1 and N2 patients) [9] and is associated with a comparable nodal relapse rate to that observed in patients undergoing radical dissection [10-12].

The applicability of the Robbins classification to the definition of radiation target volumes remains controversial. The standardization of target volumes is challenging, because the relevant anatomical-surgical references are not always identifiable on CT or MR scans. References proposed in the 1990s for the radiological definition of neck lymph node levels were inconsistent [13-18], leading to the publication of consensus guidelines for N0 patients in 2003 [19]. In 2006, Gregoire et al. [20] proposed a modification of the definition of neck regions for postoperative radiation therapy, which otherwise appears to have received little research attention.

Target volume dose-dependent toxicity (e.g., oromucositis and dysphagia) can limit the correct uninterrupted administration of adjuvant radiation therapy [21]. Therapeutic compliance would be enhanced if target volumes and, therefore, secondary toxicity could be reduced while maintaining tumor control. Standardized neck dissection by levels can yield data that allow clinicians to adapt target volumes according to the estimated risk of involvement for each node level.

## Materials and methods

For this study, we recruited all consecutive patients diagnosed with squamous cell cancer of the oral cavity or oropharynx between 2005 and 2008 who underwent surgical resection of the primary tumor, without gross residual disease, including unilateral or bilateral neck dissection with a report of histological results by neck level, and who received postoperative radiation therapy prescribed by a multidisciplinary team of head and neck specialists. Written informed consent was obtained from all recruited patients, and the trial was approved by the hospital ethics committee (Clinical Research Ethical Committe Virgen de las Nieves Hospital). Out of the 80 patients recruited, 5 were subsequently excluded after application of the following exclusion criteria: receipt of <90% of the planned radiotherapy dose (1 patient), interruption of > 2 weeks (1 patient), and the detection of cervical (2 patient), or local (1 patient) relapse during the course of the radiotherapy. The clinical-pathological characteristics of the patients in the final sample are described in Table 1.

**Table 1:** Clinical and pathological features

**Clinical**	**n**	**%**	**Pathological**	**n**	**%**
*Gender*			*Infiltration*		
Male	56	75	Vascular	7	9
Female	19	25	Perineural	13	17
*Tumor location*			*Histological grade*		
Oral cavity	58	77	G3	11	15
Oropharynx	17	23	G4	2	3
*Performance status* ECOG 0-1	70	93	*Resection margin*		
			Close	13	17
			Infiltrated	9	12
*Clinical stage*			*Pathological stage*		
I	4	5	I	-	-
II	13	17	II	-	-
III	21	28	III	24	32
IVa	30	40	IVa	48	64
IVb	7	9	IVb	3	4
*Clinical N+*	47	63	*Pathological N+*	75	100
			*Extracapsular spread*	31	41

Median age of 62 years; range, 39–78 years.

ECOG = Eastern Cooperative Oncology Group*;* N + = Positive lymph node.

Out of these 75 patients, 15 underwent both ipsilateral and contralateral node dissection. The type of dissection depended on the nodal stage, performing selective dissection in N0-N1 patients and dissection of all levels (I-V) in N2-N3 patients (Table 2).

**Table 2:** Nodal surgical procedure

**Cervical dissection (levels)**	**Ipsilateral**	**Contralateral**	**Total**
RND (I-V)	7	-	7
MRND (I-V)	33	5	38
SOHND (I-III)	30	9	39
LND (II-IV)	5	1	6
Total	75	15	90

Abbreviations: RND = radical neck dissection; MRND = modified radical neck dissection; SOHND = supraomohyoid neck dissection; LND = lateral neck dissection.

Nodal levels were intraoperatively identified following standard guidelines and then stained and delineated using surgical clips. After en bloc resection, nodal levels were coded and sent for individual histopathological study with hematoxylin-eosin staining. Adenopathies >3 mm in diameter were identified, and a topographical diagram was created of the involved levels, the number and size of positive nodes, and the presence of extracapsular spread.

Following the standard protocol, postoperative radiotherapy was administered to the site of the primary tumor (close or infiltrated surgical margin, pT4, vascular/perineural invasion, histologically undifferentiated), the dissected side of the neck (pN1 with perinodal invasion, skip metastases or inadequate dissection, i.e., total nodal yield ≤ 5, pN2-N3), and the clinical N0 contralateral non-dissected side of the neck with significant risk of subclinical involvement (considered for ipsilateral neck irradiation and/or primary in midline).

The clinical tumor volume (CTV) for elective neck radiotherapy without dissection (clinical contralateral N0) was delineated using an adaptation of consensus guidelines [19] with the above-mentioned modification by Gregoire et al. for postoperative neck radiotherapy [20]. This radiotherapy was administered to patients with at least two involved lymph nodes or with one involved lymph node generally associated with an unfavorable prognosis (at this level we only identified the infiltrated node, involved lymph nodes with a diameter >3 cm, or any extracapsular spread at any other dissected nodal level). N-pathological dissected levels adjacent to nodal levels with extracapsular spread and/or pN3 were considered high-risk levels. When the involved lymph node was located at the boundary of two levels, both levels were considered high-risk. Levels situated between two or more high-risk levels were included in the target volume, regardless of their pathological status.

According to these criteria and the estimated risk for subclinical disease, we used three types of selective nodal CTV (Table 3) and two types of standard nodal CTV (all dissected neck levels) routinely used at our center for comparison of the results.

**Table 3:** Selection of selective nodal clinical target volume (CTV)

	**Nodal CTV0**	**Nodal CTV1**	**Nodal CTV2**
Subclinical disease risk	<10%	10%-25%	>25%
Prescribed radiation dose	<35 Gy (a)	50 Gy	66-70 Gy
Levels dissected	Negative level with sufficient dissection	Negative and/or positive level without risk factors with insufficient dissection	Positive level with risk factors
	Positive level without risk factors	Adjacent negative level to nodal CTV2 (ECS and/or pN3)	
Levels not dissected	-	Ipsilateral: Non-dissected level adjacent to infiltrated dissected level	-
	-	Contralateral: Levels with risk factors	-

(a) Referred to mean dose < 35 Gy.

Abbreviations: ECS = Extracapsular spread; pN = pathological nodal stage.

The Pinnacle 8.0 version 3, 3D planning system was used, and the treatment was administered by means of a Varian Clinac-D 2100C linear accelerator equipped with multileaf collimator. An isotropic margin of 5 mm was applied to the respective CTVs to generate the appropriate planning target volume (PTV). The dose variation in a PTV was not allowed to exceed +7% or −5% of the prescribed dose. Brain, brainstem, spinal cord, lens, retina, optic nerves, optic chiasm, parotid gland, and mandible, among other structures, were delineated on the CT image. The prescribed dose to the PTV was 50 Gy to the isocenter for PTV1 and a cumulative dose of 66–70 Gy for PTV2. Concomitant cisplatin-based chemotherapy was received by 36 patients with extracapsular spread and/or positive surgical margin (100 mg/m^2^ cisplatin every 3 weeks by 28 patients and 40 mg/ m^2^ weekly by 8).

**Figure 1:**
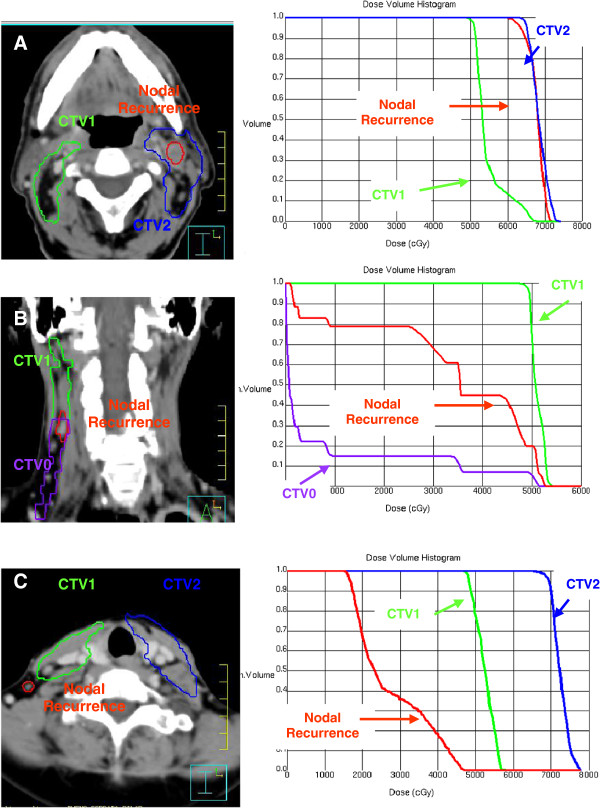
**The radiologic imaging of nodal recurrence volume was co-registered with the pre-treatment CT data set used for treatment planning.** (**A**) DVH analysis of nodal failure “in field”. (**B**) DVH analysis of nodal failure “marginal field”. (**C**) DVH analysis of nodal failure “out-field”.

Post-treatment neck CT examinations with intravenous contrast were performed at 2, 6, 12, 18, 24 months and then annually, or when a relapse was suspected. Post-treatment nodal relapse was defined by the appearance of an adenopathy in the neck. A nodal relapse detected on the CT scan was confirmed by histological study, and a new CT-simulation scan was performed using the same thermoplastic mask and parameters (isocenter, thickness and number of radiological slices) to calculate the nodal relapse volume (NRV). After obtaining the dose-volume histograms, nodal relapse was classified as: within the irradiated field (>95% of NRV within CTV1 or CTV2); within the margins of the irradiated field (20%-95% of NRV within CTV1 or CTV2); or outside the irradiated field (<20% of NRV within CTV1 or CTV2) (Figure 1).

Actuarial locoregional control and survival rates were calculated by using the Kaplan-Meier method. SPSS version 19 (IBM, Chicago IL) was used for all data analyses.

## Results

**Figure 2:**
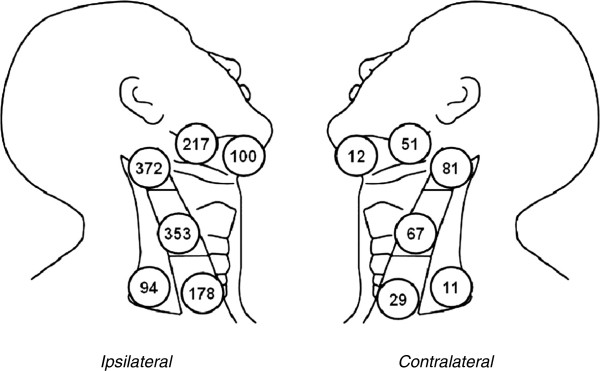
Quantification of nodal levels dissected.

As depicted in Figure 2, 1,545 lymph nodes were dissected: 1,294 nodes in ipsilateral dissections and 251 in contralateral dissections, distributed over the five neck levels. Metastasis to at least one of the examined lymph nodes was observed in all 75 ipsilateral necks dissected, which evidenced a total of 121 involved (positive) levels, i.e., a mean of 1.6 positive ipsilateral node levels per patient (1–4). These 121 levels contained a total of 183 metastatic adenopathies, i.e., a mean of 1.5 positive nodes per involved level (1–7). Level II was the most frequently involved in the oral cavity (16/31) and oropharynx (37/90) tumors; among the oral cavity cancers, level Ib was most frequent in tumors of the floor of the mouth (14/27). A regular neck dissemination pattern to levels I to III was observed in 73 (97%) of the 75 ipsilateral dissections, starting in levels Ia/Ib in 39 dissections, level II in 30, and level III in 4. In the other two ipsilateral dissections, one showed involvement of levels Ib, II and IV but not of level III and the other involvement of levels II and V but not of levels III or IV.

Only one case of metastasis to a contralateral node was detected (to levels II and Ib).

Post-treatment nodal failure was observed in 15 of the 75 patients, local failure in 9, distal failure in 7, and a second primary tumor in 3 (Table 4). All nodal relapse cases were located in the ipsilateral dissected neck. Nodal failure was within high-risk irradiated areas (nodal CTV1, CTV2) in 12 patients, in a marginal area (nodal CTV1/CTV0) in 1 patient, and outside the irradiation area (nodal CTV0) in the remaining 2 patients, who therefore did not receive post-treatment radiation therapy in this zone (Table 5).

**Table 4:** Pattern of treatment failure

**Failure**	**n (%)**	**Post-treatment months (range)**	**Prognostic factors**	**Localization (n)**
Nodal failure	15 (20)	10.2 (6–19)	Age > 62 yrs (p 0.002)	-
Local failure	9 (12)	11.4 (7–23)	Oral c. (tongue) (p = 0.042)	-
Distal failure	7 (9)	11.7 (6–20)	-	Lung (5)
				Bone (1)
				Skin (1)
Second primary tumor	3 (4)	25 (11–46)	-	Lung (2)
				Bladder (1)

**Table 5:** Failure pattern and dosimetric characteristics in patients with nodal recurrence

**Patient nº**	**Localization nodal failure**	**XRT volume**	**Failure (month)**	**Volume (cc)**	**Minimum dose (cGy)**	**Maximum dose (cGy)**	**Mean dose (cGy)**
1	II-III ipsilateral	Nodal CTV2	6	9.1	6498.2	6980.0	6602.9
2	III-IV ipsilateral	Nodal CTV2	12	3.45	6489.9	6995.9	6629.5
3	II ipsilateral	Nodal CTV1	14	2.8	4399.0	6551.2	5554.1
		(Nodal CTV2 marginal)					
4	III ipsilateral	Nodal CTV0	9	9.35	175.1	3113.2	1191.4
5	III ipsilateral	Nodal CTV1	8	1.83	5685.5	7005.2	6535.1
	II ipsilateral	Nodal CTV2	8	2.51	6652.8	6741.6	6701.8
6	II ipsilateral	Nodal CTV2	7	3.5	7009.1	7255.1	7149.8
7	V ipsilateral	Nodal CTV0	19	3.1	195.0	5050.1	2789.8
8	Ib-II ipsilateral	Nodal CTV2	8	3.35	6561.1	7171.2	6805.5
9	II-III ipsilateral	Nodal CTV0	17	2.9	589.9	5101.0	3091.0
		(nodal CTV1 marginal)					
10	Ib ipsilateral	Nodal CTV1	11	2.8	6491.0	7001.0	6682.2
	II ipsilateral	Nodal CTV2	11	1.9	4901.0	6281.2	5402.3
11	II ipsilateral	Nodal CTV2	7	9.8	6595.3	6798.4	6639.0
12	Ib ipsilateral	Nodal CTV2	7	2.9	6598.2	6792.0	6689.3
13	II ipsilateral	Nodal CTV2	8	2.1	6601.4	6850.1	6602.2
14	Ib ipsilateral	Nodal CTV2	11	2.8	6599.0	6899.4	6598.0
15	Ib ipsilateral	Nodal CTV2	10	10.2	6278.7	7195.5	6658.9

Abbreviations: CTV0 = low-risk clinical target volume; CTV1 = moderate-risk clinical target volume;

CTV2 = high-risk clinical target volume.

The dosimetric study of nodal failures revealed a mean NRV of 4.4 cc (1.9-10.2 cc). Mean, minimal, and maximal doses were, respectively, 6518 cGy, 6420 cGy, and 6894 cGy for patients with nodal failure in the irradiated field; 3091 cGy, 590 cGy, and 5101 cGy for those with failure within the margins of the field; and 2357 cGy, 320 cGy, and 4421 cGy for patients with nodal failure outside the radiation field (Table 5).

Out of the 75 patients in the study, 33 (44%) received postoperative selective nodal irradiation, with a mean reduction in target volume of 118.6 cc per patient (21.9 cc-234.7 cc), i.e., nodal CTV0, with a mean reduction of 1.8 levels per patient. Dose stratification by levels allowed us to administer dose-escalated radiotherapy from a dose of 50 Gy, only applying 66–70 Gy in high-risk levels. Out of the total of 186 irradiated levels, 40% received a high dose (66–70 Gy: nodal CTV2) and 60% a low dose (50 Gy: nodal CTV1).

After a mean follow-up of 32 months, 45 patients (60%) were alive and disease-free, 2 were alive with disease (3%), 22 patients died from the tumor (29%), and 6 died from other causes (8%). Nodal relapse-free survival rates were 84% at 12 months and 79% at 36 months.

## Discussion

Following positive nodal dissection, standard target volume radiotherapy includes all node levels considered to be high-risk by the surgeon, coinciding with the extent of the dissection and including retropharyngeal lymph nodes, which are not usually dissected [13,22]. Selective nodal irradiation hypothetically offers a decrease in target volume with no increase in marginal failures, considering the neck as a set of volumes for mapping high-risk neck regions. Evidently, selective nodal irradiation would not significantly reduce the target volume in patients with bulky metastasis and/or multiple neck level involvement.

It proved possible to administer postoperative selective nodal irradiation to nearly half of the present series of 75 head-and-neck cancer patients, obtaining a significant reduction in target volume. Dose-volume histogram analysis showed that the dose for recurrent nodal disease was comparable to or higher than the target prescription. Nodal failure was predominantly observed in the high-dose volume (CTV2), suggesting the need to discern a radiation-resistant subpopulation within CTV2.

Given the likely relationship of radiotherapy-induced toxicity with the target volume and administered dose, this toxicity might be significantly reduced by a selective irradiation approach. In the TROG 91:01 trial, which included 350 head and neck cancer patients, Poulsen et al. [23] reported that the target volume in the second phase of treatment had the strongest influence on the development of odynophagia and need for aggressive nutritional intervention (gastrotomy or insertion of nasogastric tube), finding that the likelihood of developing grade-4 (G-4) odynophagia was 36% when the volume was > 82 mm and 16% when it was <18 mm (p = 0.0001).

In our series, acute toxicity most frequently presented as mucositis and dysphagia in the patients undergoing radiotherapy (G-3 mucositis in 16%; G-3 dysphagia in 14%) and in those treated with concomitant chemoradiotherapy (G-3 mucositis in 27%; G-4 mucositis in 6%; G-3 dysphagia in 21%). Out of the 75 patients, only 1.3% received <95% of the prescribed radiation dose; the dose administered to neck volumes represented 98.4% of the prescribed dose (nodal CTV1: 99.6%, nodal CTV2: 98%), 90.6% completed their radiation therapy plan with no more than one day of interruption, and all completed the plan with less than six days of interruption; only 3.5% of interruptions were secondary to toxicity, a lower percentage than in previous reports [24,25]. In the study based on SEER’s (Surveillance, Epidemiology and End Results) [26] database, which included 5,086 patients with head-and-neck cancer, 29.6% of the patients treated with surgery and radiotherapy showed treatment discontinuity and/or non-adherence (33.3% of those with oral cancer and 33.5% of those with oropharyngeal cancer).

The development and incorporation of postoperative selective nodal irradiation techniques into clinical practice requires the routine performance of neck dissection by level. The possibility that the results may be inferior to those obtained with en bloc dissection was refuted by Upile et al., who found no difference in total yield between specimens sent to the histopathologist en bloc or divided into levels [27].

The variability in the number of nodes obtained from neck dissection can only be partially attributed to anatomical differences among patients, given that previous studies indicated a minimum of 28 nodes on each side of the dissected neck [28]. The primary cause of nodal variability is generally related to the surgical technique. Although the boundaries of radical dissection are defined in consensus guidelines [7,8], they may be modified by surgeons based on their own professional experience or because the terminology used to define the different types and dimensions of neck dissection is inconsistent. A substantial reduction in nodal variability can be expected if surgeons strictly adhere to the anatomical boundaries established in consensus guidelines for dissection procedures. The number of nodes is usually lower in selective procedures, such as supraomohyoid or lateral dissection, due to the smaller number of dissected levels [9-12]. The amount of lymphoid tissue dissected for histological study by level should always be the same, regardless of the type of dissection (radical, modified, or selective). In the present series, there was a minimal variability in the mean number of nodes identified in each level as a function of the type of dissection.

The histopathological report plays a key role in the assessment of the dissected specimen. The standard procedure for studying a dissected node after hematoxylin-eosin staining is to remove a longitudinal section for examination under light microscopy to detect metastatic deposits [29]. These partial findings are then extrapolated to the remaining nodal volume. Although an incontrovertible diagnosis would be provided by examination of 5-μm sections of the whole node, as in sentinel-node techniques, this is a laborious and costly procedure, and the clinical relevance of the detection of occult subpathological metastatic deposits remains unclear [29].

Radiotherapy oncologists have devoted considerable research efforts to measures for the protection of normal tissues from radiation, with a focus on minimizing their exposure. However, there have been few attempts to determine the target volume for irradiation in each specific situation, and no standardized patterns of lymph node relapse after postoperative neck radiation therapy have been established, which may be because target volumes generally include all lymph nodes on both sides of the neck. To our best knowledge, the present investigation offers one of the few descriptions of a selective nodal irradiation protocol for patients undergoing surgery for oral cavity and pharyngeal cancer according to the estimated risk for each nodal level.

## Conclusion

In conclusion, nodal involvement in oral cavity and pharyngeal cancer follows a predictable and gradual pattern across the different neck levels, supporting the selective neck irradiation approach. The availability of a reliable postoperative nodal-involvement pattern by level allows clinicians to determine target volumes according to the estimated risk for each nodal level. The neck failure pattern obtained after a postoperative selective nodal irradiation protocol does not differ from that obtained after a standard irradiation protocol. Nodal recurrence is most frequently detected in high-risk nodal levels receiving high irradiation doses. The target volume with selective nodal radiotherapy is significantly lower than the target volume with a standard nodal radiotherapy protocol.

Selective nodal irradiation of the neck is not yet a standard treatment. However, greater understanding of the natural history of the tumors and the availability of increasingly sophisticated radiotherapy techniques will facilitate further trials to support oncologists in the optimal selection of high-risk target volumes.

## Competing interests

The authors declare that they have no competing interest.

## Authors’ contributions

MMC conceived and developed the project and supervised RT applications. IML participated in this design and coordination. MMC, ITM, IML and RDA collected the data. JMRAR critically reviewed the manuscript. All authors read and approved the final manuscript.
